# (Remembering) Vector Coding of Boundaries and Objects in the Subiculum

**DOI:** 10.1002/hipo.70074

**Published:** 2026-02-16

**Authors:** Colin Lever

**Affiliations:** ^1^ Department of Psychology University of Durham Durham UK

## Abstract

This review offers a personal and historical perspective on spatial representations of the local environment in hippocampal regions CA1 and subiculum, as derived from extracellular electrophysiological recording of neurons in these regions in freely behaving rodents. I focus upon geometric responses and discrimination learning in CA1 place cells, and upon boundary vector cells, boundary‐off cells, and vector trace cells in the subiculum. Vector trace cells are a type of boundary vector cell with an additional memory capability.

## 1 Introduction

This review offers a personal perspective based on imperfect recollections of my and others' work on spatial representations of the local environment in regions CA1 and subiculum. The review is selective, for example touching only relatively briefly upon topics such as theta, exploration, novelty, and anxiety, in favor of a more unified story of vector coding in the cells of the rodent subiculum. The focus is on geometric responses and discrimination learning in place cells (Lever, Wills, et al. 2002), our discovery of boundary vector cells (Lever et al. 2009), boundary‐off cells and boundaries as inhibitors of spatial firing (Stewart et al. 2014), and vector trace cells (Poulter et al. 2021), a seemingly distinct subtype of boundary vector cell which shows memory for vectors. I try to add wherever possible memories relating to John O'Keefe and his lab in UCL, where I worked in various roles from MSc student to post‐doc from 1995 to 2005.

As we pretty much all know, but can barely mitigate, remembering is not an act of revealing, but of reconstructing. From continuous experience, we select a few choice vignettes, which may more likely suggest dives to pearls of prophecy and insight, rather than the grubby accumulations of accident and toil. Even for modest accomplishments, this is probably no less true. I am also pretty sure my episodic memory is below average: my apologies are obligatory.

## 2 The Route to John O'Keefe: Knowing Where and Getting There

My first encounter with what was explicitly John O'Keefe's work was brief—perhaps 5–10 min long—in 1993/4. As part of what became a conversion to neuroscience, I was studying ‘The biological bases of behaviour’, part of Birkbeck College's Diploma in Psychology. The tutor, John Harrison, briefly described an experiment that recorded from neurons in the rat brain which involved what we would now describe as cue manipulations and cue removal. I thought it was a rather interesting approach to simultaneously record individual neurons and behavior while altering the stimuli around the rat, and at the end running what John would have called a probe trial. Harrison asked the class a question about how many cues needed to remain to preserve the firing, which I and a couple of others answered correctly. He spoke positively of John O'Keefe and the experiment. I am not sure I gave it that much conscious thought later on, but it must have stuck with me.

Wanting to feed my curiosity further, but having no passionate interest in any of the other Psychology modules, I applied for UCL's MSc in Neurological Science. Although the timing is not clear to me, I’d looked at a UCL prospectus relating broadly to the MSc (and other degrees), noticed John O'Keefe's name, and connected it up to the experiments briefly described on the Birkbeck course. Despite various other London options, I only applied to UCL. Having done no science since A level maths, Prof Maria Fitzgerald, the Course Convenor, kindly constructed a conversion course for me consisting of 1st and 2nd year BSc Neuroscience courses (1994/5). Despite strong concerns that I would be ‘an eternal student’, my parents kindly paid for the first year. I loved the courses and was awarded an MRC studentship for the MSc (1995/6).

## 3 A Small‐Scale Model of External Reality

A serious difficulty in getting an MSc project lined up was that as long as John's lab was a theoretical possibility, there was an automatically low ceiling upon my enthusiasm in working with anyone else. John was far from keen on getting MSc students in, and my skill set (see below) must have seemed especially useless. I browsed through other options, asked around, but remained keen for John. Why exactly? My reconstruction suggests these factors were key: (1) I really liked the level and approach John took, as per my Birkbeck module introduction; (2) I was very curious about how memory worked, and John worked on memory. (3) John was suggesting that the rodent hippocampus generated an explicit model of the external world, which I found compelling. I think this was and remains a key motivating principle for me, and it is of course relevant to Subicular vector coding.

I had been to a very inspiring talk by a biologist given in 1988/9. It was part of an Evening Talks club (name forgotten) run by (now Lord) John Krebs at Pembroke College, Oxford, where I was an undergrad (second year), studying English Literature (1987–1990). I think I arrived late from my digs in Bullingdon Road, so missed the speaker's name. The talk was called something like ‘Models of the world’. Fairly early in the talk was a simple metaphor, (elaborated later in 1995), about how the clothes on a washing line modeled the wind's direction and magnitude. This progressed to the idea using the movements of birds flying and nesting on a cliff to construct a model of the cliff itself, and then to the climax—the idea of the bird's brain modeling the cliff and local environment. Here, I think he alluded to how there was already some evidence for this kind of representation in the rat hippocampus. I recall that many audience questions indicated more interest in other, more famous ideas of the speaker, who was Richard Dawkins. At the risk of boring the reader with fussy reconstruction, this weak memory gains good plausibility because: (1) Dawkins and Krebs were frequent co‐authors and good friends; (2) the ideas behind Krebs' paper (Krebs et al. 1989) on larger hippocampi in food‐storing birds, of course citing (O'Keefe and Nadel 1978), would have been pregnant in Krebs' mind at that time (Krebs spent much research effort on this theme, e.g., see (Clayton and Krebs 1994; Biegler et al. 2001) and several others). Here are excerpts from Dawkins' later 1995 version: *“Having built in the capacity to simulate models of things as they are, natural selection found that it was but a short step to simulate things as they are not quite yet—to simulate the future. This turned out to have valuable consequences, for it enabled animals to benefit from “experience,” not trial‐and‐error experience in their own past or in the life and death experience of their ancestors, but vicarious experience in the safe interior of the skull.”* (Dawkins 1995). I came across similar points when reading Arthur Koestler (1964), one of my favorite books (on creativity, curiosity, novelty) on my road towards neuroscience, quoting from Craik's *The Nature of Explanation: “if the organism carries a “small‐scale model” of external reality and of its own possible actions within its head, it is able to try out various alternatives, conclude which is best of them, react to future situations before they arise, utilize the knowledge of past events in dealing with the present and future…”* (Craik, 1943, quoted in Koestler 1964, 506).

John's various comments to dissuade me included things like: “It's not possible for you to do electrophysiological recording as a Masters project”; “I guess you could do anatomical tracing. Do you know what that is?”; “There won't be any behaviour”; (since I didn't know how science worked): “You do realise you don't get me as a supervisor. You will get a post‐doc.” And so on. Somehow, my intuition said—Agree to all these downgradings of expectations. I kept coming back. Eventually, I heard from Maria that John had relented. As it turned out, I loved anatomy. The cross‐species similarity of mammalian brain regions and wiring helped me understand why so many neuroscientists study rodents.

Finishing the MSc, I had a PhD studentship waiting for me in another lab that I was keen on, but of course was not with John. I episodically remember staying up all night (literally), thinking how I could turn it down. I held out. I remain immensely grateful to the Head of Anatomy Dept, Prof Geoffrey Burnstock, who said “well it doesn't seem fair to stop him now surely”, and awarded me a department‐allocated MRC PhD studentship to work with John. As regards my parents' concern re eternal studenthood, I told them, omitting any mention of being a PhD student, that John had offered me a low‐paying job as a research assistant.

## 4 To Remap, or not to Remap

It seems important to begin with remapping, for example, (Kubie and Ranck Jr. 1982; Muller and Kubie 1987) for several reasons: it was very important to the lab at the time; it is key to thinking about what Boundary vector cells do; John's thinking about remapping led, in the end, to the Boundary vector cell (BVC) model (discussed further below). The fantastic, pioneering work of the Brooklyn lab was ever‐present in the O'Keefe lab. We are all indebted to that Kubie and Muller simplification (Muller et al. 1987): the plain walled cylinder, the foraging task, the Kubie white card, and quantification of location and direction. See (Kubie 2025; Nadel 2025; Taube 2025; O'Keefe 2025; Fenton 2026) for some of that history. I would love to have read a Bob Muller equivalent.

I later asked John if Bob Muller could be my external Examiner for my viva, which was largely great fun. To paraphrase Lowell on Berryman—few voices now stick in my ear as Bob's. Passionate. Abrasive.

John told me he wanted to find a point where the system broke (I don’t recall his exact words) from representing the map for one environment to generating a different environment's map. John described the (O'Keefe and Burgess 1996) *Nature* paper as ‘what you can learn from an interesting failure.’ The expected state transition, that is, the remapping, whether it would be from a small square to a large square or from square to elongated rectangle, did not occur. Instead, most of the place cells fired in a broadly predictable manner across the four rectangular boxes, for example, in the south‐west corner of all four boxes. Neil Burgess came up with an elegant boundary vector model to explain that predictability (O'Keefe and Burgess 1996). Place cell firing could be explained by summation and thresholding of (up to) four Gaussian tuning curves, each peaking at a particular distance from, and in a direction perpendicular to, one of the four walls of the rectangular box. For example, one place cell might be explained by inputs peaking at short distances to the west and south walls (Neil had stuck printouts of rate maps of all the cells in (O'Keefe and Burgess 1996) on his office walls in the O'Keefe lab. That has partly inspired my ongoing conviction that I must know my lab's datasets on a cell‐by‐cell basis). At this point, the boundary vector model was not explicitly cellular. That came later: Burgess et al. 2000; Hartley et al. 2000; Lever, Burgess, et al. 2002; Barry et al. 2006. The idea was that a place cell's place field reflected input from a few boundary vector cells (BVCs). One BVC might fire maximally to a boundary 10 cm to the north of the subject, another BVC to a boundary 30 cm to the east, and so on. To anticipate for a moment, I seem to recall that my finding of place cell field doubling upon insertion of a barrier during my PhD (1998 or 99), see (Hartley et al. 2000; Lever, Burgess, et al. 2002; Lever, Wills, et al. 2002), as predicted by the (O'Keefe and Burgess 1996) model, was an important fillip to Neil's BVC modeling. The first author used an exclamation mark in describing the result in (Burgess et al. 2000).

So the next test John wanted to explore along this line of ‘find the point of transition between map 1 and map 2’ was given to me: to investigate the influence of shape by comparing square versus circular walled environments (I started electrophysiology in 1997, one year into my PhD (1996–2000), once John called me into his office regarding my continued anatomical work and said ‘We need closure; do you want to carry on with anatomy or do electrophysiology?’. ‘Electrophysiology’, I said. So that was that). The Muller/Kubie story implied that the circular‐to‐rectangular box remapping they had obtained in 1987 was due to shape: “[T]he firing pattern of a [place] cell in an apparatus of one shape could not be predicted from a knowledge of the firing pattern in the other shape.” (Muller and Kubie 1987).

## 5 Remapping Not by Shape

I first did some preliminary work in three rats that was pretty persuasive, at least to me, that the remapping I found between square and circular shaped boxes was not based on shape but on position in the room. Lacking the access at this stage to the large cue‐controlled environment (CCE) lab that I later had as my own for several years, I did what I could in the smaller, narrower, lab adjacent to John's office where John had conducted the (O'Keefe and Burgess 1996) experiments, and where John and Mike Recce did linear track work. I placed the rat in two separate morph box configurations, a square and cylinder. The morph boxes were early ones designed by Neil Burgess, and made by him and Clive Parker, John's crossword‐loving electronics technician. Each morph box had 32 separate pieces, configurable as, for example, a square with 8‐piece sides, or as a quasi‐circle with a 32‐piece circumference, with 50‐cm high walls. Albeit with few cells, I obtained complex/global remapping (Brooklyn/Trondheim terms) across the two standard configurations of square and circle, and then changed each configuration to the other shape. The 2 × 2 matrix of firing rate maps of each cell for room position versus shape showed convincingly that room position was the critical determinant. I then did a further test trial series probing for room position effects (Kate Jeffery might have suggested doing a room‐position probe?). In slow incremental steps (~15–20 cm at a time), I gradually moved the circle towards the square, recording at each increment. As the circle got closer to the standard square position, it became even clearer that the remapping was not caused by shape differences, but room position. Crucially, when I placed the circle such that it was centred identically to that of the standard square (well past midnight at this point), the place cell pattern was very similar to that of the initial square (and of course very different to that of the initial circle). None of this was published, though I showed the room position probe in my SFN poster (in 1999/2000?).

As I recall, John and Neil had two key pointers regarding the square‐circle research. (Point 1) *Ensure that head direction is anchored across the differently‐shaped environments* (Neil's particular emphasis), that is, that HD‐sense resets were not triggering remapping. Once I got to having a larger lab, I was confident that my lab set‐up delivered this anchoring well (Lever, Wills, et al. 2002), and I did similar procedures in the (Lever et al. 2009) study. Indeed, it was difficult at the end of the multi‐day experience trials to get the rats' cells to rotate 90° CCW in a rotation probe. I had to not only rotate the external cue card, but also rotate the entry point from over the south wall to over the east wall. Each of these alone was insufficient. I think it's much easier to ‘fool’ the head direction sense of a mouse than a rat (For the importance of entry point, see (Sharp et al. 1990)). (Point 2) (A later point, after I’d done the preliminary position‐vs.‐shape work above). *Look at early exposures*. I was particularly happy to do this, because I was (and remain) very interested in the related themes of curiosity, novelty, exploration, and encoding. So I was determined to ensure that I would capture the *very first* seconds and minutes of experience in the environments.

One thing I did *not* want to do was keep using the morph boxes for the long‐term trial series (e.g., up to 22 consecutive days). The idea of this multi‐day experiment which exposed the rats to alternating square‐walled boxes and cylinders, day after day, was to observe the possible effects of experience upon firing patterns in these square and circle shapes. Practically speaking, reconfiguring the morph‐square into a morph‐circle would have taken too much work in the inter‐trial intervals if the morph box was the standard environment. In my view, the inter‐trial interval was to be devoted as much as possible to thinking (e.g., of future manipulations) while smoking on the outside balcony (facing Gower St) past the histology room. The good old days. So I got the workshop to make two fixed squares and two fixed cylinders, and I decided to use the morph box just for probes after the consecutive‐day long‐term trial series.

Given the intended emphasis of this review, I will cut a long story short here, and simply say that my PhD work went on to show, in the published main paper (Lever, Wills, et al. 2002), and its supplementary information sheet 1 (Lever, Wills, et al. 2002), that initial exposures to square and circular boxes which differed only in shape did not elicit complex/global remapping. This gave further support to the BVC model approach to thinking about place cell spatial firing patterns.

However, with rat‐specific days/weeks of experience, incremental remapping did occur. Furthermore, importantly, the probe trials that I conducted showed that the now‐remapped patterns showed a lot of transfer by shape, especially from original square to morph square (fig. 4, Lever, Wills, et al. 2002), and circle patterns transferred not only to morph circles but to classic even‐sided octagon morphs (unpublished). John asked me to train and supervise Tom Wills, and Tom ran a further two rats, which became rats 4 and 5 of fig. 4 (Lever, Wills, et al. 2002), showing shaped‐based firing pattern transfer. This remap and transfer‐by‐shape series helped to set up the paradigm for the pattern completion by attractor dynamics experiment in (Wills et al. 2005). I also did the wiring, surgery and screening for the high‐yield rat in that study which showed the attractor drift modeled by Neil, getting it ready for the clever, intermediate‐octagon probe trial series that Tom had designed. To my knowledge this study remains a classic demonstration of ideas in Marr and Hopfield, and I am proud of my contribution to that. Simply put, we found good evidence for the idea that memories are attractor states of neuronal representations. Marr (1971) had suggested that the extensive recurrent connections in CA3 could support an auto‐associative memory, whereby partial input could produce retrieval of an entire stored representation, and Hopfield (1982) invented a simple model of this process involving the minimisation of energy states. The (Wills et al. 2005) study was also an influence upon me in that it forced further thinking about the idea of systems switching between encoding versus recall, which continued to take me, inter alia, towards Mike Hasselmo's SPEAR models (reviewed Hasselmo 2025).

## 6 Progress is a Journey with Obstacles En Route

As mentioned above, my work had given even more impetus to the BVC model approach to thinking about place cell spatial firing patterns. Beyond the convenience with which the BVC model explained place cells, might there actually be physiological BVCs in the hippocampal formation? In looking for a new direction after (Lever, Burgess, et al. 2002; Lever, Wills, et al. 2002), I recall I was looking at either the Entorhinal cortex or Subiculum for spatial firing correlates that were not context‐specific, like those of CA1. I first targeted the Entorhinal cortex. I think I did two implants targeting the Entorhinal cortex. I don’t recall the co‐ordinates. Both implants failed. The electrodes had some gliosis muck on them, perhaps exacerbated by too‐closely juxtaposed tetrodes. Two complete failures were annoying, so I turned to the subiculum. I’d long been intrigued by Pat Sharp's idea of a ‘universal map’ in the subiculum (P. E. Sharp 1997, 2006, 2025). Sharp was emphasizing how the subiculum appeared to “transfer a single, abstract representation from one environment to another” (P. E. Sharp 1997), which could be seen even while CA1 cells were remapping those environments. I think we would now say that many subicular cells don’t ‘remap’, but some certainly do. There is much to say about how subicular firing is surprisingly unlike what you might expect to see if you approach it as the ‘fourth synapse’ (CA1‐Subiculum) after the trisynaptic loop (Ento‐DG, DG‐CA3, CA3‐CA1), but I have to skip that here. Sharp had commented upon subicular firing being shaped by boundaries. As I recall, I went into the subiculum with at least three things in mind, this issue of non‐remapping, boundary‐related firing, and the relative timing of CA1 and subiculum (P. E. Sharp 1997, 1999), and with the general motivation that the subiculum was not well‐studied compared to CA1. That remains true.

There were a few obstacles. I don't think anyone had implanted two chronic unit‐recording drives in different regions in the O'Keefe lab before me, or at least not in my time there. At any rate, getting successful implants from one drive in the subiculum and one in CA1 turned out to be relatively straightforward. Much more problematic in practice was the reduced ability to isolate the firing of any single subicular pyramidal cell. I'd heard informally that Matt Wilson had a go at the Subiculum, presumably from a sleep and systems consolidation angle, and he was frightened off by the lack of good isolation. I understood his concern as I was familiar with his warnings about the potential dangers of imperfect isolation (Quirk and Wilson 1999). However, I accepted that some analyses would be off limits and carried on. Another great obstacle was the glacial slowness then of what we'd now call the pre‐processing. It was not just the isolation but also the higher numbers of spikes. Compared to CA1, it took eons to load a trial and to cut the cells. I think once for a particularly busy set of subiculum tetrodes it took 45 min to transfer the trial from the recording computer to the analyzing computer. I could spend an entire weekend cutting a six trial sequence on just one tetrode. I might record 8 putative cells on a Subiculum tetrode and isolate just two. With the same technology I might get up to 15 CA1 cells on one tetrode, *and* isolate them all *and* do it quicker. The effort/cell‐yield ratio in Subiculum was appalling compared to CA1. However, Subicular spatial fields were beguilingly different to that in CA1, and I started to realize I could not leave the Subiculum alone (so it has proved). Another initial obstacle was that John was actually not especially keen on me recording from the Subiculum. However, characteristically, he did not stop me as such, and also characteristically, as soon as John saw the very different and seemingly mathematical patterns (e.g., emphasized in Abad‐Perez et al. 2025) in the rate maps from my first subicular implant which he too found beguiling, he actively encouraged me to keep going.

**FIGURE 1:**
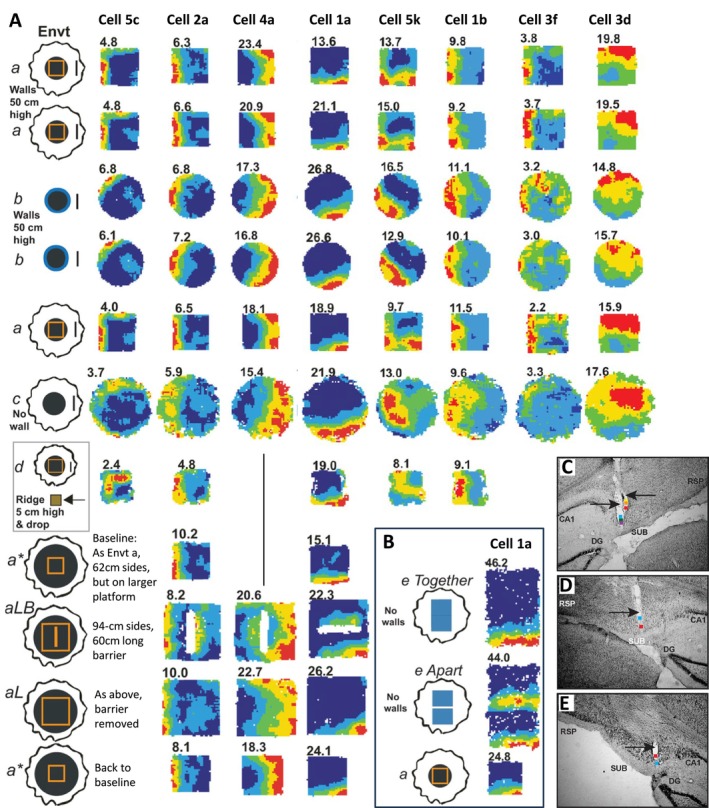
Examples of the initially‐reported Boundary Vector Cells (BVCs). (A) Each column shows a single BVC's firing over different trials in different types of environment. Simultaneously recorded CA1 cells showed global remapping across environments a, b, c (e.g., ensemble in Figure 2). (B) Further cell 1a trials on two open platforms tightly juxtaposed (‘Together’) or separated by a 13 cm gap (‘Apart’). Rate maps for open platform environments c, d, e include regions where rats peered over platform edges. Cells 5c & 2a were initially shown in (Barry et al. 2006). Number top left of each rate map is peak rate (Hz). (C–E) Red squares show recording sites for cell 5c (C), cell 2a (D), & cell 1a (E). Firing rate map methods & cell number labels are as per (Lever et al. 2009), from which this figure is adapted.

**FIGURE 2:**
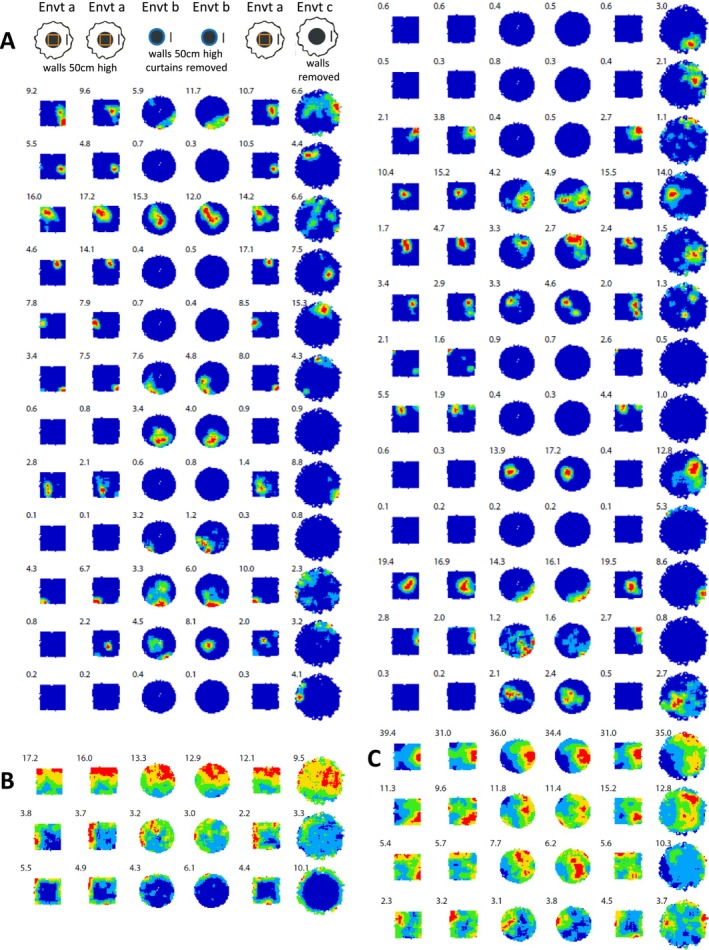
Concurrently with CA1 place cell ensemble showing ‘global’ remapping across different environments, some subicular cells show boundary‐related generalization. 25 CA1 place cells (from three tetrodes) (A), simultaneously recorded with 7 subicular pyramidal cells (from three tetrodes) (B, C). Each row shows firing rate maps for given cell over 6 trials in three distinct environments a, b, c (same as in Figure 1). Note generalization in cells in (B): Top two cells are BVCs ‘3d’ and ‘3f’ in fig. 3 in (Lever et al. 2009), & Figure 1 (this paper); bottom cell fires along the environments' perimeter, less so in Envt b. Number top left of each firing rate map is peak rate (Hz). From (Lever et al. 2009) dataset.

My intention was to elicit the most violent (the kind of phrase I used) remapping from CA1 that was possible across three different environments centred identically in lab space (see section 5 above), while simultaneously anchoring the head direction system across those environments. I suspected that this might not be trivial to implement, so I was very pleased on the technical point that I succeeded in eliciting what we now call global remapping in all the rats with CA1 cells while anchoring head direction. Importantly, I was confident of the value of a wall‐less (drop) environment being one of the three environments, having previously observed strong remapping across walled to wall‐less (drop) environments (e.g., fig. 1C in Lever, Wills, et al. 2002). This turned out to be crucial to persuading myself that I had found BVCs. Figure 1 shows examples of some of the initially reported BVCs, that is, that I presented in (Barry et al. 2006) and (Lever et al. 2009). It was striking that BVCs could respond to walls and drops similarly (e.g., see Figure 1, compare environments a & b versus c & d), and immediately so, upon exposure to novel environments containing them (Lever et al. 2009; Stewart et al. 2014). This was all the more striking because walls and drops elicit rather different sensory and motor patterns (discussed further below). Moreover, strikingly, observing such similar vector‐type responses across environments which CA1 place cells remapped so strongly (Figure 2) greatly added to my respect for the rather complex and abstract job that BVCs were performing.

There was no single Eureka moment for discovering BVCs because their character cannot be fully confirmed with a single trial. Mini‐Eureka moments that I remember semantically were first observations of firing similarity in some subicular cells across walls and drops, first without CA1 cells (March 2003, my first subicular rat was a double subiculum implant) and then, importantly, with simultaneous observation of complex/global CA1 remapping (July 2003).

## 7 Eliciting Additional Vector Fields

The more joyous Eureka moments came with two ways of eliciting additional vector fields. It is perhaps interesting to consider why they were more joyous. I think it relates to the idea of experimental control, and in turn to understanding. If you can manipulate a situation to generate extra firing fields in the exact locations and orientations that you predict, then it seems you have some useful understanding of the phenomenon in question, which is exciting. If one likes to please others, there is also the point that Neil was always wanting barriers to be inserted to elicit a field doubling, so there was also the pleasure of validating his BVC model, done as a probe trial in the same CCE‐lab room, once I had done the carefully controlled novelty‐then‐familiarization trial series.

The first method was as per Neil's suggestion for hippocampal place cells, that a barrier should induce a second field in a region predicted by its wall field (Lever, Burgess, et al. 2002; Lever, Wills, et al. 2002). Examples of this for BVCs are shown for BVCs 2a, 4a, and 1a in Figure 1. Exactly as predicted. Tick.

**FIGURE 3:**
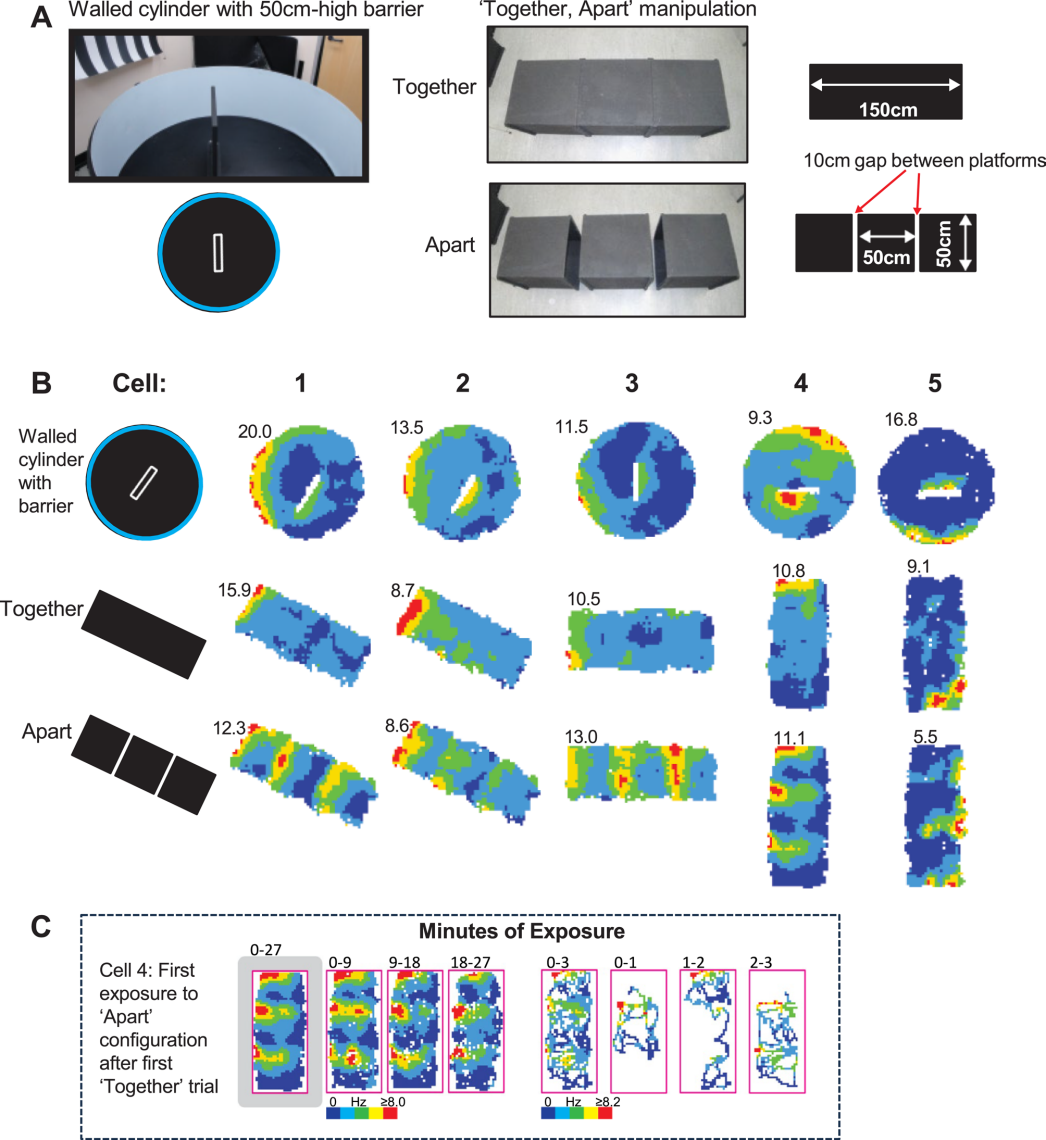
Together‐apart manipulation. (A) Testing environments. *Left*: Standard walled cylinder (50 cm high wall) used as baseline environment, but with 50 cm high barrier inserted testing vector field repetition by walled boundary. *Right*: ‘Together, apart’ manipulation testing vector field repetition by drop boundary. (B) Five BVCs (each column one cell) recorded from two rats in 2010 and 2011. Additional vector field elicited by drops in ‘Apart’ configuration as well as by walled barrier in cylinder. (C) Drop‐elicited firing fields are present immediately upon first exposures to drops. Number top‐left of rate map: Peak rate (Hz). Adapted from (Stewart et al. 2014).

Even more delightful was the novelty of showing field repetition by creating extra drops in the ‘Together, Apart’ manipulation using two tables (Figures 1B, 3). This was not one of Neil's suggestions, but I felt the results (first done in August 2005 in my CCE‐lab room) were particularly convincing as validation of his model. Why so? John's lingo was ‘once you think you have discovered a phenomenon, you need to throw rocks at it to try sink it.’ One thing I took from John is that the best rock is often another manipulation in another trial. It does depend on your claims for a cell, but I often feel that you can’t just shuffle your way to classifying a cell, and/or apply statistical models based on very few trials or indeed just one. To me at least, the ‘Together, Apart’ manipulation was a particularly useful rock to throw because a drop is a very different kind of boundary to a wall, entailing different motion and sensation. With a drop, rat exploration typically takes the form of ‘peering over’ the drop; with a wall, exploration often involves rearing on hind legs, often up against a wall, a classic response to environmental novelty which fascinated me from the moment I saw it (I wrote the only review of rearing to date in (Lever et al. 2006), where I tried to dissociate spatial learning vs. emotional contributions to rearing). Sensorily, the somatosensory and auditory input is also rather different at the boundary: in a walled box, the floor and walls are juxtaposed, but in a wall‐less environment there is (almost) no vertical surface. Furthermore, the wall‐less drop environment in my lab set‐up afforded a proximal and distal view onto a new visual lab‐world, compared to that afforded by the square‐walled and circular‐walled environments.

I had two potential explanations to try to rule out about the fields being similar at drops and at walls in the circular environment (see environments b and c in Figure 1). Although it might not seem especially likely, one explanation of the field constancy across these two environments (which CA1 cells globally remapped, Figure 2) was that these similar firing fields were in a broadly similar position in lab‐space. Whether as the sole or a key factor in combination, ideothetic and/or allothetic cues relating to room position could be contributing to that similarity. My second concern, anticipating our later discovery of Vector Trace cells (Poulter et al. 2021), was that the drop‐elicited field (in environment c in Figure 1) could reflect memory of the wall field (in environment b particularly, but also environment a, in Figure 1). Initial exposures were always to the walled box.

These concerns were already partly ruled out by recording some BVCs on the holding platform outside the Cue Controlled Environment (CCE). I purposely encouraged or permitted behavior such as resting on the holding platform to be different from that in the testing environments inside the CCE Environment, and never did the foraging task on the holding platform. So it took a bit of effort to run those trials showing that these BVCs had fields on the holding platform obeying the same preferred vector tunings as that within the curtained CCE, despite being located about two metres away centre to centre, see environment d in Figure 1. But then, ok they were similar, but were these just similar *by chance*? So there was a particular joy in the field repetition experiments; they really quietened down my internal critics voicing the above alternative explanations. Of course, I wanted to just do a couple more such manipulations…

The editors call for comments as regards initial publications: a couple of points here. (1) We first reported BVCs in (Barry et al. 2006), a multi‐author paper with various types of contributions with three PIs, John O'Keefe, Kate Jeffery, and Neil Burgess. As I (with Neil) had been to the 2004 conference in Japan on which the Special Issue in *Reviews of the Neurosciences* was based, the task of deciding authorial order in this multi‐author paper (never a fun task) fell to me, and I found it rather awkward, that is, self‐serving, to make myself be the first author. This has led to misunderstandings. John Kubie and I think others thought that Caswell Barry had initially discovered BVCs. It was one of those multi‐author papers where no author, except presumably the very first and last named, is entirely satisfied. Seeing the cells' rate maps in black and white but from color not greytone originals did not help; it hurts me viscerally to look at those BVC rate maps. (2) I was slow with publishing these findings. As well as moving cities, setting up a new lab, and beginning IVF, I wanted, in a John O'Keefe type way, to throw rocks at a few more cells. The Mosers' publication of border cells (Solstad et al. 2008) forced our hand, so we rushed out the (Lever et al. 2009) report. Reception‐wise, I note its citations seemed to take a leap from 2013 to 2014. Why? Potential factors were: the ‘Space in the brain’ Royal Society conference we held in 2013, the special issue that emerged (reviewed, Hartley et al. (2014)), and the Nobels to John and the Mosers. Overall, it's worth noting that BVCs provide a strong example of a computational hypothesis being tested.

## 8 Maps Are Not Made Under Siege

One task suggested to me was to describe John's lab (location: 4th Floor, Anatomy building, Gower Street, UCL) during my time there (1995–2005), and thus John, further to the *en passant* snippets above. John was the first leader I respected. I would say he led more by example, than verbal instruction, more David Beckham than Roy Keane, to pick the one‐time captains of Manchester United (This imperfect analogy comes to mind because John greatly admired how Beckham executed solutions to complex 3‐D spatial problems in real time). One aspect of John's example was the clarity of his priorities, the first being fundamental discovery. John was obviously uninterested in the material trappings of success, and exhibited near‐zero interest in the managerial roles that might aid their acquisition. John did take teaching relatively seriously, however. The most important thing to me, and a defining feature of John's leadership style, was that John tried as far as possible to implement the approach implied in a sentence in John/Lynn's Preface to (O'Keefe and Nadel 1978): “Maps are not made under siege.”

There are of course many routes to accelerating discovery. One might be ringing up post‐docs at unsociable hours demanding project updates. That was not John. John encouraged, prodded, suggested lines, but never harassed. He used to say the best thing was to give a student a problem, and let them get on with it, such that the student/post‐doc took ownership of it. One thing he said was ‘the ideal thing is when the student thinks they thought of it’. At least at that time, John was not a ‘manager’ in almost any sense of that word. When I went to the Blanchard lab in January 2005, I saw for the first time weekly ‘lab meetings’, led by Bob Blanchard, which everyone was supposed to attend. This was a professional managerial enterprise, such as might occur in a small company, or group in a company. This is something to do with size of course. The Blanchards had many undergraduates and MSc students, which was not the case in the O'Keefe lab then. John would occasionally take on 2–3 medical students doing an undergrad project, but would generally leave them to a PhD student or post‐doc, like myself or Tom Wills, to supervise (Of an almost‐hilariously unsuccessful attempt to get rats to do different behaviors in squares and circles, I will say nothing here).

This is not to say we did not get together. In the O'Keefe lab, we had several ad‐hoc ‘journal club’ type discussions and many ad‐hoc informal discussions, often with John there and contributing. These were great fun and very instructive for understanding what a good experiment was, what good controls might consist of, the presence or absence of a key probe manipulation, and so on. We also had discussions about politics. John would also host parties at his nearby flat for Xmas, Vivas, and so on, usually with his excellent homemade houmous. Later, not so fun, I recall us all sitting down together to discuss the fact that John's MRC programme grant was rejected again after resubmission and there being some implications of that to discuss. When money was running out in the early 2000s, John asked us to repair things wherever possible and not buy so much and to check with him if we wanted to buy something that cost more than one/two hundred pounds. These were the closest things that came to lab meetings, but as for a regular weekly or monthly meeting, I don’t recall anything like that. The objective of our assembly was generally for insight, not resource management. Personally, that was exactly how I liked it. Having done 9–5 jobs in regular workplaces, I loved being left to get on with things. Arguably, with the greater demands of chronic extracellular Ephys, this approach works best with committed, competent, and emotionally resilient students. Like a mouse in a Morris watermaze, you had to ‘sink or swim’ was the phrase.

I am very grateful to Jim Donnett and Kate Jeffery, both post‐docs when I entered the lab, and Patrick Martin, who was finishing his PhD, who all contributed to my training. John asked me to train Francesca Cacucci and later Tom Wills. John Huxter had already set up a basic recording lab in Canada, so he didn't need much from me. I like to think that I helped set up a nurturing environment in the O'Keefe lab. Although I set them only on the beginning of their journey, it's great that Francesca and Tom stayed in the field and have done so much to understand the early development of spatial neurons in rat pups, and thus on causal relationships, from (Wills et al. 2010) to (Muessig et al. 2024) more recently on BVCs in the subiculum. For other accounts of (different epochs in) the O'Keefe lab at UCL, see (O'Keefe 2025; Jeffery 2025; Burgess 2026).

## 9 Anxiety, Theta, Novelty, Exploration

I went on a Bogue Fellowship to the Blanchards lab in 2005 for 5 months. My main goal was to learn more about exploration, via my side‐obsession with rearing on hind legs, and find out about the extent to which rearing reflected anxiety motivation, as distinct from being a mechanism for obtaining spatial information, and combining allothetic and ideothetic information (Lever et al. 2006; Poulter et al. 2018). Appendices to Gray and McNaughton (2000) written by McNaughton touching on rearing spoke about the Blanchards pioneering ‘risk assessment’, so I wanted to go to the source. Another goal, which I emphasized to John, was developing an anxiety‐involving task to explore spatial cognition (That didn’t work out: use of cats is a non‐starter in the UK, even just as stimuli to scare rats). John kindly continued to cover my salary while I was there. This experience enlivened a new side‐interest in anxiety and theta (Pentkowski et al. 2006; Wells et al. 2013; Levita et al. 2014; Pervolaraki et al. 2019; Bindi et al. 2022; Hines et al. 2023), and my long‐standing interest in novelty and exploration.

All these themes came together later, sparked by reading an early version of Neil's model of grid cells (Burgess 2008) in the bath after moving to Leeds. Briefly, as concerns this story, Neil related the slope of the frequency to running speed relationship to type 1 theta mechanisms, with slope inversely controlling grid scale, and suggested that the y‐intercept of the frequency‐speed relationship related to type 2 theta and arousal. A rather different approach to theta was in Gray and McNaughton (2000)'s theory of the anxiety‐generating hippocampus, within which Neil McNaughton (see McNaughton (2025) review) showed repeatedly that different classes of anxiolytic drugs reduced the frequency of theta generated by stimulating the reticular formation. So, given some relationship between arousal and anxiety, and at any rate with a cognitive and emotional dissociation in mind, I wondered if we could combine the two models to make the prediction that anxiolytic drugs would reduce the theta y‐intercept, as well as test Neil's prediction that since grid scale increases in novelty, spatial novelty would be associated with reduced theta slope. We nicely confirmed these predictions (Wells et al. 2013; Korotkova et al. 2018; Hines et al. 2023), which have been replicated by others, for example, (Newman et al. 2013; Monaghan et al. 2017). This dissociation seems rather provocative to me. Reduction of theta y‐intercept from systemically administered anxiolytic drugs, while observing animals freely behaving is surely rather useful assay for testing anxiolytic drugs, but seems to be rather uninfluential. I am also interested to explore our hypothesis that theta frequency reduction in novelty serves encoding (Hines et al. 2023).

## 10 ‘Boundary‐Off Cells’ and Boundaries/Objects as Inhibitory

**FIGURE 4:**
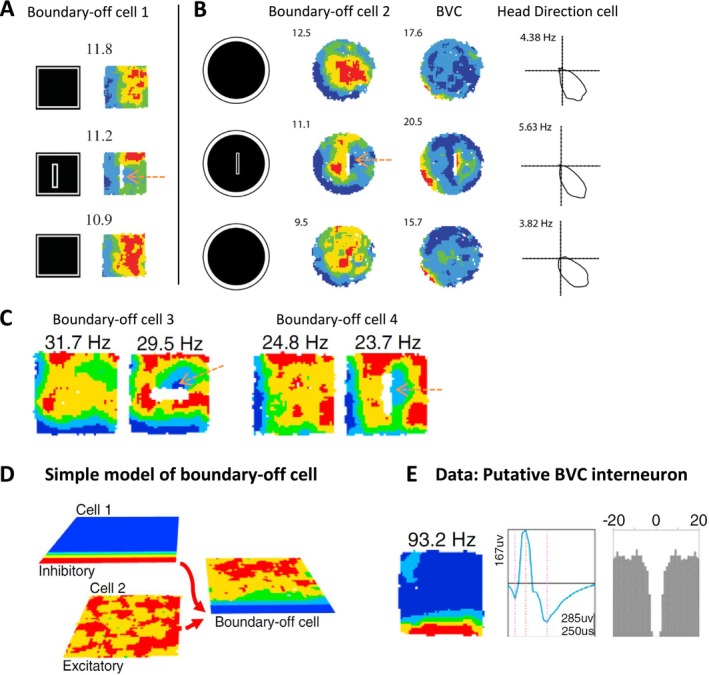
Inhibition of firing by boundaries. (A, B) Examples of first‐reported ‘Boundary‐Off’ cells. Top and bottom rows show baseline trials, middle row shows inhibiting effects of barrier insertion. Pink dashed arrows point to zones of inhibited firing. (B) Shows three simultaneously recorded cell types: A Boundary‐off cell, a BVC, and a Head direction cell, showing head direction sense is well anchored across trials. Insertion of barrier elicits a new field east of barrier in BVC, and simultaneously inhibits firing east of barrier in Boundary‐off cell. (A, B) Adapted from (Stewart et al. 2014). (C) Further examples of Boundary‐off cells. Note, for example, north‐east orientation of barrier‐elicited inhibited firing zone in cell 3, consistent with inhibitory ‘boundary‐to‐south‐west’ tuning (minimal firing along south and west walls). (D) Model: Boundary‐off cell for example, showing minimal firing along the south wall can simply be modeled as combining excitatory input across entire environment (cell 2), together with inhibitory input from BVC interneuron with ‘boundary‐to‐south’ tuning (cell 1). (E) Example of (probable) BVC interneuron that the model posits. The very high firing rate, and the relatively high amplitude and long peak‐to‐trough interval of cell's waveform compared to axonal waveforms, strongly suggests cell is an interneuron. (C–E) Adapted from (Poulter et al. 2018).

I moved to Leeds University in September 2005. I have had lots of good and bad luck. One of the bad pieces of luck I’ve had is experiencing lots of lab shutdowns after leaving UCL, typically refurbishments to meet animal care guidelines. We had to undergo three periods of refurbishments at Leeds University's psychology department which closed my lab each time, and three at Durham too, some very long‐lasting. Two at Leeds occurred during Sarah Stewart's PhD with me there, one lasting around a year. So we weren’t able to get as much recording done as we hoped, but one crucial insight was into the inhibitory, as well as excitatory, effect of boundaries. One of the overall goals was to elicit additional vector fields by various cue manipulations. I have already mentioned additional drop‐boundary elicited fields in the ‘Together, Apart’ manipulation (Figures 1B, 3). Interestingly, Sarah and I noticed that barriers did not just elicit additional fields in BVCs; they very clearly inhibited firing in other cells. In particular, we noted a lawful inhibition in a cell type we called the ‘Boundary‐off cell’ (Stewart et al. 2014; Poulter et al. 2018). Boundary‐off cells have preferred angular and distance tunings just like BVCs, and look like inverse BVCs (Figure 4A–C). They can probably be explained by a simple model whereby a cell receives two types of inputs: excitatory cell(s) firing fairly ubiquitously throughout the environment, and inhibitory cell(s) of the BVC‐like pattern (Figure 4D). This model makes a clear prediction: there should be subicular interneurons which are BVCs. It's not always trivial to distinguish interneuron‐specific signals, but the example in (Figure 4E), recorded by Steven Poulter when he was my post‐doc, is pretty convincing.

Because recording time was curtailed in Sarah's PhD, we did not report many boundary‐off cells in (Stewart et al. 2014). This may have contributed to the neglect of what I think is a very interesting and functionally important phenomenon in the hippocampal‐entorhinal system, though it has sparked aspects of some grid cell models, for example, (Krupic et al. 2014; Widloski and Fiete 2014). Actually, boundary‐off cells are rather numerous in the rodent subiculum, a point Steve often emphasizes. They respond, like the entire subicular vector system, to ‘objects’ as well as ‘boundaries’ (see discussion below). None of this can be appreciated in empty environments. Furthermore, importantly, boundary‐off cells are not the only cells in which inhibition occurs. One can often see inhibition in BVC firing fields, where there is inhibition from the direction opposite to that of the given BVC's angular tuning (one of a few features suggestive of an attractor system).

## 11 The Subicular Vector System: ‘Objects’ as Well as ‘Boundaries’

**FIGURE 5:**
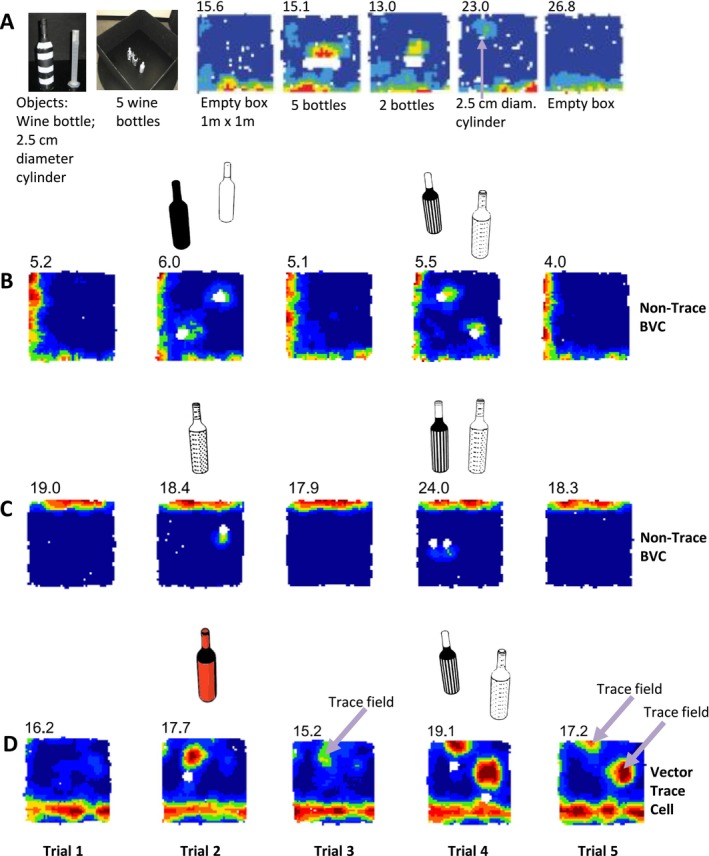
BVCs including VTCs respond to ‘objects’ as well as ‘boundaries’. (A) Early example (recorded 2011) of BVC generating an additional vector field in response to closely‐juxtaposed wine bottles (five or two), and also to a small object: A 2.5 cm‐wide measuring cylinder shorter than a wine bottle (unpublished, Stewart and Lever). Light purple arrow points to object location. (B–D) Examples of two non‐trace BVCs (B, C) and one VTC (D) generating vector fields in response to individual wine bottles as well as boundaries (50 cm high box walls). Light purple arrow points to trace fields. BVCs including VTC show no obvious signs of distinguishing object identity. All data from rats. Each row depicts one cell over five trials. Number top‐left of rate maps: Peak firing rate (Hz). (B–D) Adapted from (Poulter et al. 2021).

The other disappointing effect of the lab shutdowns during Sarah's PhD in Leeds was that we weren’t able to do much recording with objects. My feeling was that the Subicular vector system would need to be responsive to objects as well as boundaries, and that to a BVC there would be likely no discrimination between them except that of geometrical extent. Any such discrimination would seem to call for an additional mechanism. John O'Keefe used to say that an ‘object’ was quite an advanced concept for a rat. Sang Ah Lee emailed me when I was in Leeds and I think we chatted re the landmark versus boundary systems, and how BVCs might respond to landmarks. One crucial motivation for me was an idea still not tested: to use the same cues in boundary‐response and landmark‐response type experiments so that if any differences arose, they could not be attributed to the materiality of the cues. For instance, one possibility would be to test the extent to which a BVC would be impervious to the difference between a bottle used in a classic additional‐field manipulation, a bottle that consistently predicted a reward location or turn towards reward, and one which never did. The starting point was to generate additional fields in the locations predicted by the BVC model, but now using objects, not just walled barriers and drops. Sarah's preliminary data showed that what rodent hippocampologists would consider ‘objects’ did indeed elicit additional fields in a lawful manner, with even a noticeable response in the rat to an object only 2.5 cm wide (example, Figure 5A).

Importantly, using smaller cues such as wine bottles to elicit additional vector fields has key advantages. Notably, observing the predicted additional field does not require such a large environment. Relatedly, leveraging the high predictability of the location and extent of the predicted additional field, one can place the object in such a way that the predicted field should not overlap with any existing firing fields, at least for the exemplar BVC(s) under particular consideration. This greatly adds to the certainty that an additional field was generated specifically by the cue. Another advantage is that one can insert more than one cue within a trial, both together (Figure 5A) and, often more interestingly, apart (Figure 5B–D). Obtaining two or more additional vector fields within the environment in the predicted locations illustrates the degree of experimental understanding and control of the BVC phenomenon (Figure 5B–D). This degree of predictability and control is not yet possible with ‘landmark vector cells’, hippocampal place cells that have landmark vector responses (Deshmukh and Knierim 2013). In advance, you don’t know which place cells are going to have landmark vector responses, nor what the preferred distance and angular tunings are going to be for a given cell. To my knowledge, this reduced experimenter's predictability also applies to the Entorhinal object vector cells, which are reported to respond to objects but not extended surfaces like walls (Høydal et al. 2019). You can it seems predict the object vector field only after one object trial. In contrast, a BVC has both boundary‐driven fields and object fields, and inspection of a BVC's boundary‐driven field predicts its object‐driven properties pretty well.

## 12 Episodic‐Like Memory?: Vector Trace Cells

I emphasize these points because it seems not a coincidence that the eureka moment for Vector Trace cells (VTCs) came exactly from this level of experimenter control using one, then two, wine bottles (Figure 5D). Simply put—what else could explain the findings? Steven Poulter, my post‐doc, came to work with me and for a while Sang Ah Lee after his PhD work on spatial learning in the Morris water maze (e.g., Poulter et al. 2019). Anthony McGregor's water maze room was just a few metres down from my recording lab in Durham's Life Sciences unit. In the first cue trial shown, Steve inserted a wine bottle such that the expected additional cue field would occur in a region of minimal firing near the north‐west of the box (Figure 5D, trial 2). Actually, there was a trace field in the next trial after the bottle was removed (Figure 5D, trial 3), but what with several cells and tetrodes to look at, or perhaps with no time to assess the data in the inter‐trial interval, there was no eureka. Steve then inserted two different wine bottles in a second cue trial: one bottle a little north of the earlier placement, and another in a south‐east position (Figure 5D, trial 4). The additional cue‐elicited fields were exactly as expected, with high firing rates. He then ran a post‐cue trial 5 after removing the bottles, then loaded the trial and cuts for trial 5. At first, Steve assumed he had incorrectly loaded the previous trial 4, as the rate map for this cell showed two bottle‐elicited fields that were very similar in terms of position and field firing rates. He then reloaded trial 5, checking the trial label he specified was correct, but saw the same rate map as he did before! That was Steve's eureka moment (October 2015). The obvious explanation in the absence of the bottles was something new in the BVC world: memory.

**FIGURE 6:**
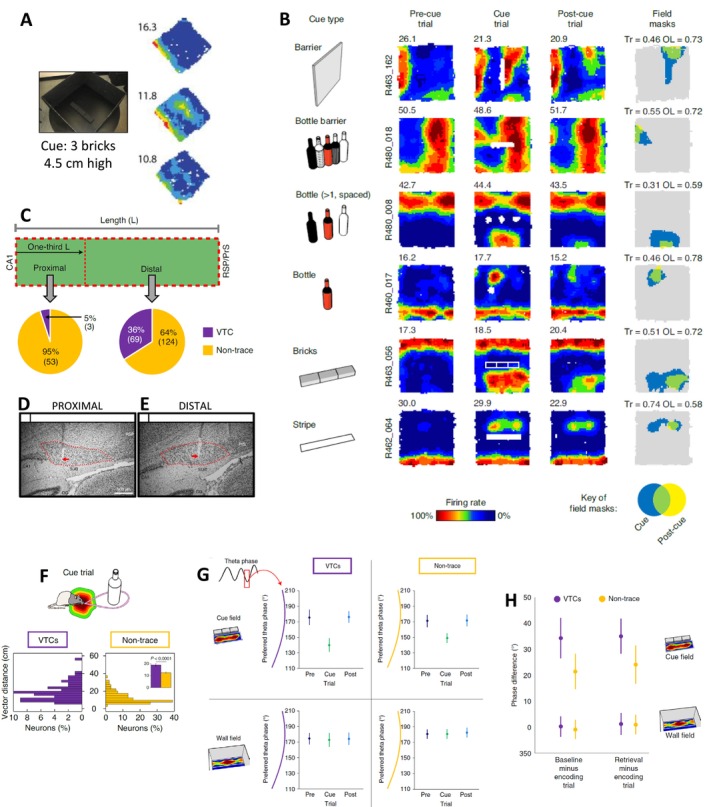
Vector trace cells (VTCs). (A) Early (2010/11) unquantified hint of VTC phenomenon. Inserting 3 bricks elicits additional vector field in predicted location, which leaves weak trace when bricks are removed (2010/11, unpublished, Stewart and Lever). (B) Examples of VTCs showing strong memory for vector to different types of cues. Right column: Values of trace score (Tr) and cue/post‐cue field overlap (OL) used to quantify traces. (C–E) Subiculum was divided into proximal (near CA1) and distal subregions: VTCs were much less common in proximal (D) than distal (E) subiculum. (F) Distance component of the vector to cues was greater in VTCs than non‐trace BVCs. (G) Both non‐trace BVCs and VTCs show earlier theta phase of firing in the cue field, but not the wall field, and only in the cue trial. This earlier‐going shift is significantly stronger in VTCs than non‐trace BVCs, suggestive of an encoding marker (H). (B–H) Adapted from (Poulter et al. 2021).

There was much more work to do of course including odor controls, and excellent work on quantifying the traces by Tom Wills, but that cell and those bottles in those trials were fundamental. Sarah and I had seen some hints of vector traces before (e.g., Figure 6A), but the clarity of these new traces was on another level, and Steve and I felt we were onto something. VTCs show one of the most unambiguous demonstrations of memory in the hippocampal system to date. The rapid learning after just one exposure trial of first one cue location and then two other cue locations suggests VTCs might encode spatial elements of episodic memories. This idea will have additional support if, as we suspect, vector traces are context‐specific. Such considerations are for another time. Figure 6B from our report of VTCs (Poulter et al. 2021) shows examples of the VTC phenomenon using different cues, with quantifications of the trace score and overlap, two measures developed by Tom Wills to classify VTCs, and thus to discriminate them from non‐trace BVCs.

Lesser eurekas fell to me. Briefly: (1) I’d classified the subicular recording sites into proximal subiculum (aka ‘prosubiculum’) and distal subiculum (aka ‘subiculum’). Once we’d got some quantification of which cells were VTCs, I sat on my bed late one night with a pen marking off proximal and distal into a spreadsheet and gradually came to realize with anatomy‐hat joy that the VTCs were nearly all in the distal subiculum (Figure 6C–E) (Interestingly, VTCs show longer distance‐tunings than non‐trace BVCs (Figure 6F), and this remains true for distal‐only cells). (2) Relatedly, the distal subiculum and proximal subiculum showed different mean phases of firing with respect to the theta oscillation, with distal cells being about 60° earlier (Poulter et al. 2021), lending further weight to the idea of functional differences across the proximodistal axis. Irregularly spaced tetrodes and the extracellular ephys approach more generally are not optimal methods to determine anatomical locations. So I was very pleased when (Kitanishi et al. 2021) using linear probes replicated our results rather closely, showing that cells in layers of the distal subiculum fired on average ~60° earlier than in proximal subiculum. A third additional finding needs some background. Having long been fascinated by how the memory system negotiates the different needs of encoding, such as during novel scenarios, and pattern‐completive retrieval, I have always found Hasselmo's SPEAR models very inspiring (Hasselmo et al. 1996, 2002; Hasselmo 2025). I had found that some exemplar cells that learned to discriminate the two shapes in the (Lever, Wills, et al. 2002) dataset, showed different mean theta phases of firing before stopping firing in one shape. The popularity of the (Mehta et al. 2002; Harris et al. 2002) approaches, emphasizing a simple conversion from instantaneous rate to phase, meant I knew I would encounter skepticism about the causality of phase.

Much later, consistent with Hasselmo's SPEAR cholinergic (Hasselmo et al. 1996) and theta phase (Hasselmo et al. 2002) models, but also giving a consistent direction to encoding and retrieval in CA1, we showed a pretty convincing pattern of results: consistent with need for encoding, novelty elicited a *later* phase of firing in CA1 place cells (closer to the pyramidal layer peak), and scopolamine blocked this later phase‐in‐novelty shift, and induced even *earlier* mean phases in a familiar environment, which gradually reverted to the usual mean phase as the drug wore off (Lever et al. 2010; Easton et al. 2012; Douchamps et al. 2013). This was encouraging. Given that VTCs offered such a clear cellular memory correlate, and thus a straightforward delineation between encoding (cue field in cue trial) versus retrieval (trace field in post‐cue trial), they provide a test of the (Hasselmo et al. 2002) model. The results, albeit not quite what I predicted, were consistent with the model. Upon insertion of a cue, both VTCs and non‐trace BVCs showed an earlier theta phase of firing, which occurred only in the cue trial (encoding trial), and only in the cue field (i.e., not in the wall field). See Figure 6G, which illustrates the specificity of this encoding‐related phase change (Though space restrictions prohibit laying out all the partial, background evidence, an *earlier* phase of firing in the subiculum is consistent with Entorhinal‐biased streams mediating encoding of novel information, and a later phase with CA1‐biased streams, mediating e.g., CA3‐driven products of retrieval). Furthermore, excitingly, this earlier‐going theta phase of firing shift was greater in VTCs, which retained traces of their fields, than in non‐trace BVCs, which did not (Figure 6H). This felt like an important insight. Steve and I are excited to consider how theta phase precession modulates this story (Poulter et al. 2023). Is it earlier phase at entry to the cue field, or phase precession, or both, which predict the memorability of the cue field? VTCs seem to offer the spatial component of an episodic memory. Will trace fields show a hallmark of episodic memory, context‐specificity? It seems appropriate to end here, full of itchy questions.

## 13 Looking Backward and Forwards

It's been 30 years since I first entered the O'Keefe lab, and thus the world of hippocampal recording of neurons and oscillations. I have enjoyed testing predictions derived from theory, and also enjoyed accidents. Both these routes to discovery seem crucial. Consistent with the advocacy of neuro‐ethology in O'Keefe and Nadel (1978), it's useful to generate avenues whereby what's inside the brain can teach you what's important to find out. Re theories, most provocative to me are computational models which can be used to generate novel experimental predictions about future data in perhaps limited domains, rather than those which seek to maximize accommodation of a large amount of existing data.

Looking forward, science leaders might pay more attention to the possibility that PhDs, and likely post‐doc posts, can worsen mental health, but it's not an easy problem to solve. Myself, I had great fun as a PhD student and post‐doc. The post‐doc role can offer the serious playfulness of a protracted adolescence without the adult responsibilities of running a lab, negotiating academia, etc., but the precarity of the post‐doc (which has barely improved in 30 years) is a high cost. In a world where the difference between big and small labs gets bigger and the insane wastefulness of detailed, fully‐costed grant applications with routinely low success rates gets worse not better, it seems increasingly necessary to retain childish joys in finding out new things, big or small, to build mental resilience, and to have bucketloads of optimism bias.

## Funding

This work was supported by the Biotechnology and Biological Sciences Research Council (BB/G01342X/1 and X/2; BB/T014768/1; BB/M008975/1).

## Data Availability

No new data was generated for this review paper.
